# Data on optimal operation of Safarud Reservoir using symbiotic organisms search (SOS) algorithm

**DOI:** 10.1016/j.dib.2020.105327

**Published:** 2020-02-26

**Authors:** Aliakbar Rezaei-Estakhroueiyeh, Navid Jalalkamali, Mehdi Momeniroghabadi

**Affiliations:** a Water Resources Management, Department of Civil Engineering, Kerman Branch, Islamic Azad University, Kerman, Iran; b Department of Water Engineering, Kerman Branch, Islamic Azad University, Kerman, Iran; c Department of Civil Engineering, Kerman Branch, Islamic Azad University, Kerman, Iran

**Keywords:** Evolutionary algorithm, Safarud reservoir, Operation, Symbiotic organisms search algorithm

## Abstract

This data article explains the time-series data for optimal operation of Safarud Reservoir located in Halilrood basin in the south of Iran for a period of 223 months, from October 2000 to April 2019. The utilized data included the release of the reservoir, reservoir inflow, reservoir storage, evaporation and precipitation. A model based on Symbiotic Organisms Search (SOS) algorithm was also developed for the optimal operation of Safarud Reservoir. The analysis of the objective function showed that the best solution achieved by the SOS algorithm was 10.89. Also, the analysis of these datasets revealed that the SOS algorithm was efficient for the optimal operation of the reservoir problem.

**Table:** Specifications Table

Subject	Water Resources Planning Management
Specific subject area	Hydrology and Water Resources Management; Evolutionary Algorithm
Type of data	Table, Figures and Graph
How the data were acquired	Raw data were obtained from the Regional Water Company of Kerman, and the data analyzed were obtained from the MATLAB software.
Data format	Raw and analyzed datasets
Parameters for data collection	• Reservoir characteristic parameters (e.g., minimum and maximum reservoir storages, water release, and downstream water demands); • The monthly time series of reservoir inflow, release of the reservoir, water demand, evaporation and precipitation.
Description of data collection	The used datasets were provided by the Regional Water Company of Kerman.
Data source location	Safarud Reservoir located in Halilrood basin in the south of Iran (56° 58′ E longitude, 29° 15′ N latitude).
Data accessibility	All datasets are available in the supplementary file attached to this article.

**Table:** 

**Value of the Data** • Data on the release from the reservoir, reservoir inflow, reservoir storage, evaporation, precipitation and downstream water demand in Safarud Reservoir provide an overview of the operation of the reservoir from 2000 to 2019. • These datasets can be employed to analyze the status of water resources in Safarud Reservoir operation. • The dataset will be beneficial for modeling purposes related to Safarud Reservoir. • The analysis performed here with SOS algorithms solver can be adopted by other researchers for comparison. • Other researchers can use the SOS algorithm to solve other problems with certainty.

## 1 Data

Halilrood basin is situated in Kerman Province, southeast of Iran. It is located in Hamoon Jazmoriyan catchment, between Barez Mountains to the north and Makran Mountains to the south. About 70% of this basin is composed of mountainous regions, and the rest is a plain. The climate of Halilrood basin is extremely hot in summer and of moderate temperature in winter.

Halil River stretches for some 390 km running in the Baft, Jiroft, and Kahnuj districts of Kerman Province. This river is subject to periodical flooding. There is a possibility of river floods in large catchment areas due to the long duration of rainfalls. The river floods predominantly occur in mountainous regions and are discharged with a high peak and low based time. A characteristic of river floods in large catchments in semi-arid areas is their intense dissipation along the main river. In the upper parts of the river route, water penetrates the soil and enriches underground water sources. There is also fertile soil and abundant groundwater, especially around the Jazmoriyan, which provides suitable conditions for cultivating crops.

Safarud Reservoir is located in Halilrood basin (Fig. 3). In this data article, the symbiotic organisms search (SOS) evolutionary algorithm was first developed in the optimization of Safarud Reservoir operation aiming for water demand management. This problem is a challenging, realistic problem that can offer new insights for water resources planners and managers as Safarud Reservoir is a strategic reservoir in Iran. We described how we successfully optimized the operation of this strategic reservoir by a robust SOS algorithm. To this end, the objective function was defined as the minimization of total deficit for the time series datasets. The time series dataset of Safarud Reservoir includes release, inflow, storage, evaporation, precipitation, and downstream water demand for a period of 223 months, from October 2000 to April 2019. The datasets are presented in Fig. 2. Release is a volume of outflow from Safarud Reservoir (MCM). Inflow refers to the volume of inflow to Safarud Reservoir (MCM). Storage is the volume of storage of the reservoir (MCM). Also, evaporation is the depth of evaporation from the area of the reservoir (mm). Precipitation refers to the depth of precipitation in the area of the reservoir (mm). Finally, demand is the volume of downstream water demand of Safarud Reservoir (MCM).

**Fig. 1:**
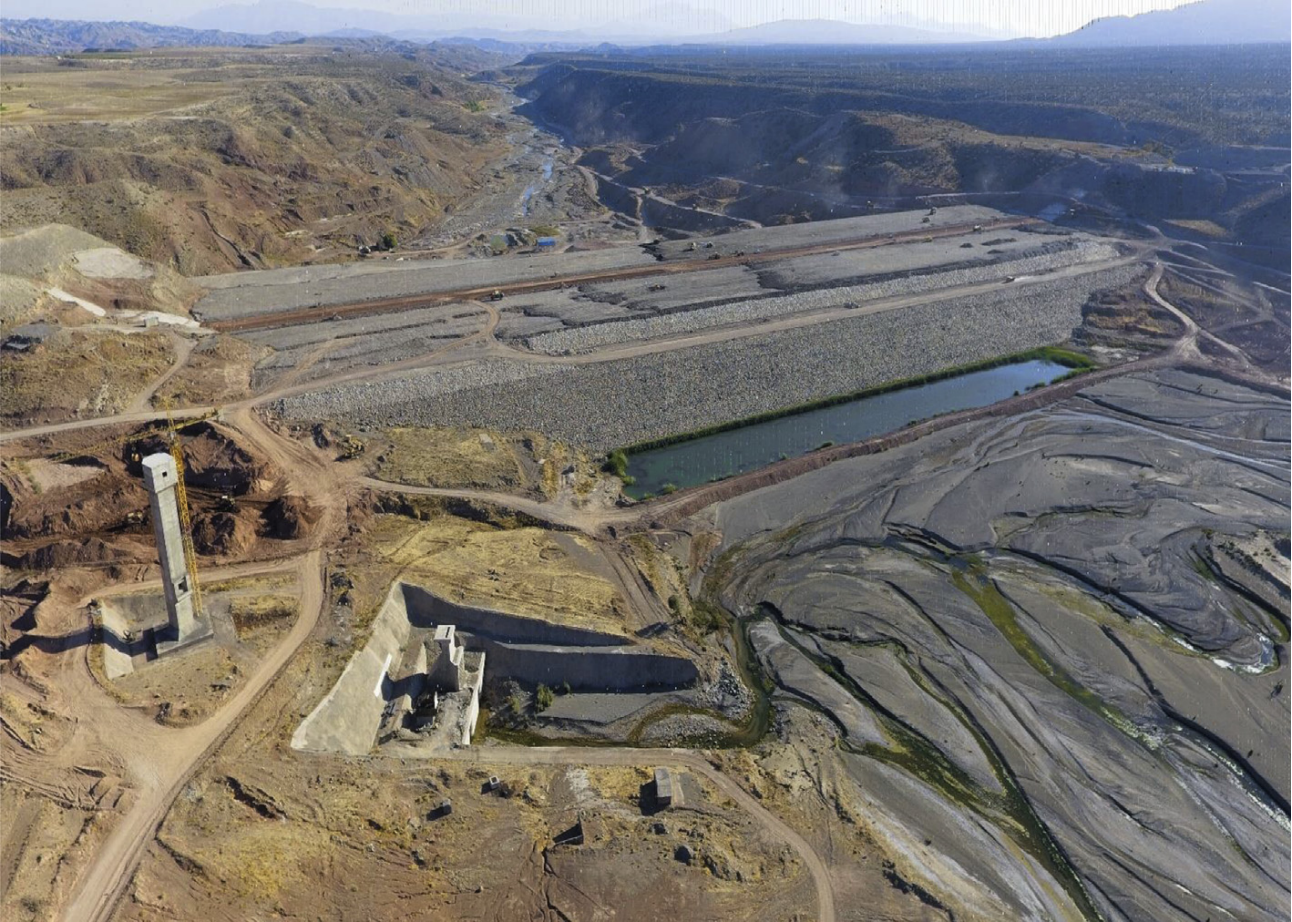
The image of Safarud dam.

**Fig. 2:**
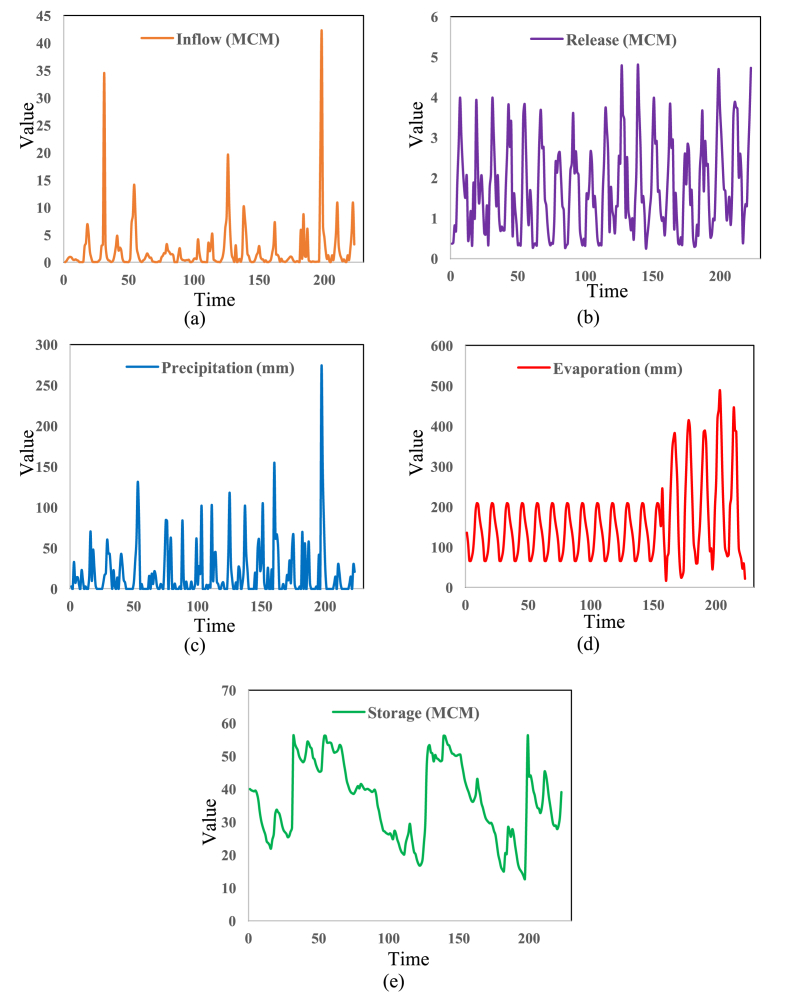
Time series diagram of Safarud Reservoir datasets, a) Inflow, b) Release, c) Precipitation, d) Evaporation, e) Storage.

**Fig. 3:**
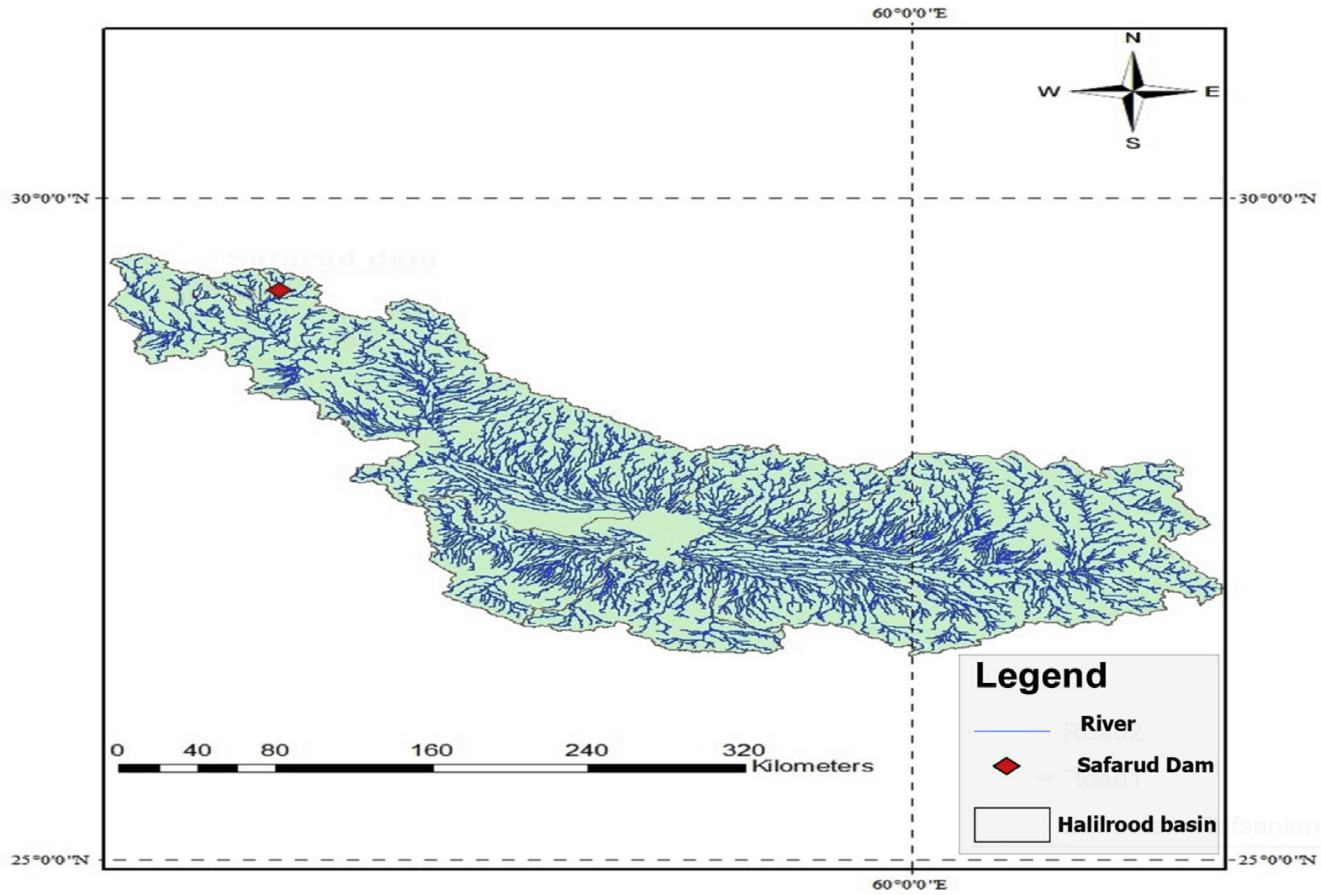
Geographical location of Safarud dam in Halilrood basin in the south of Iran.

**Fig. 4:**
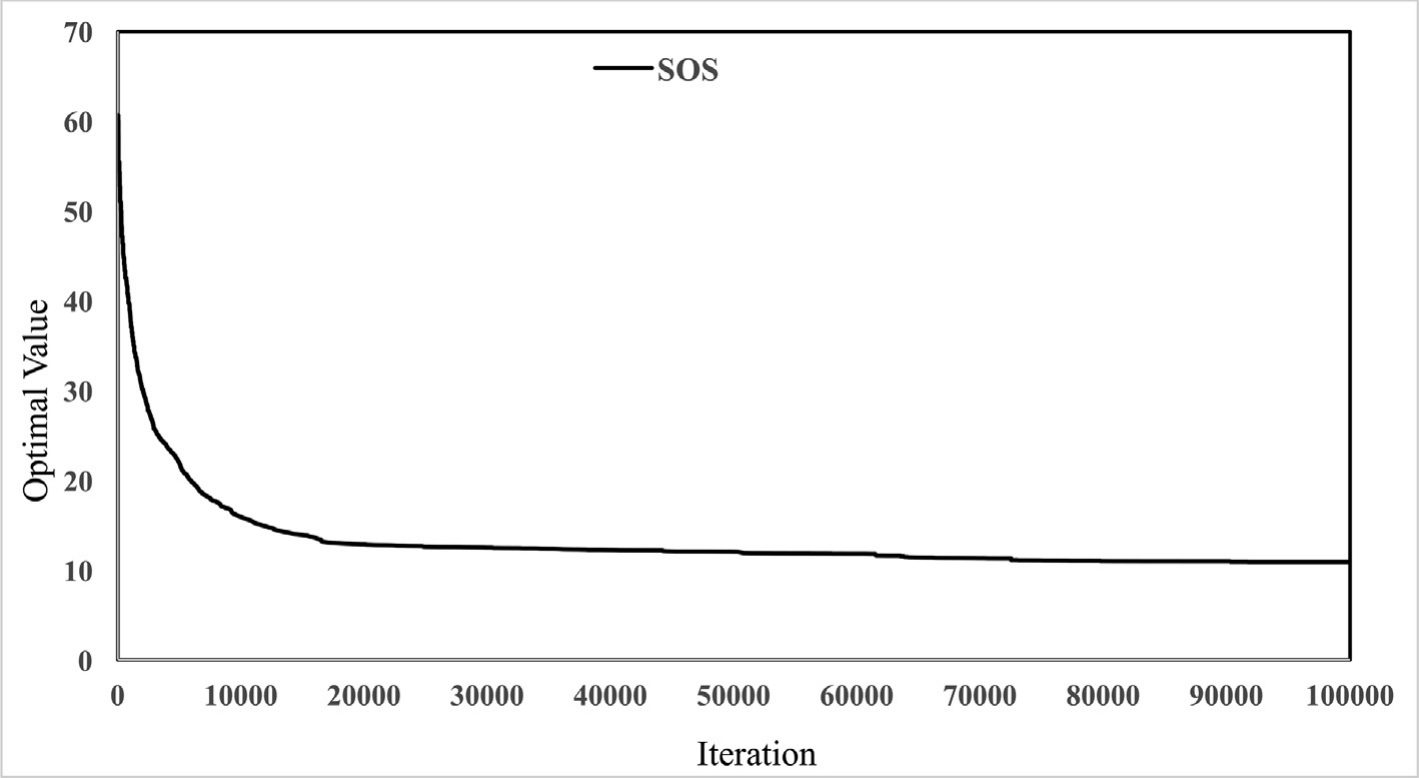
The convergence of SOS algorithm in Safarud Reservoir.

**Fig. 5:**
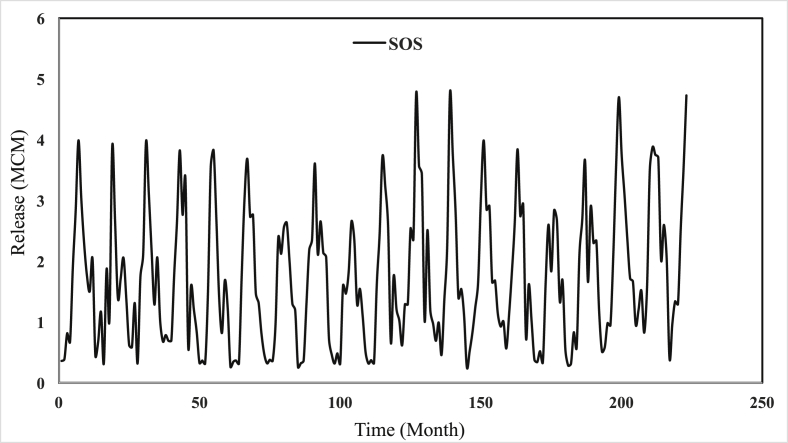
Water release patterns of SOS algorithm in Safarud Reservoir.

**Fig. 6:**
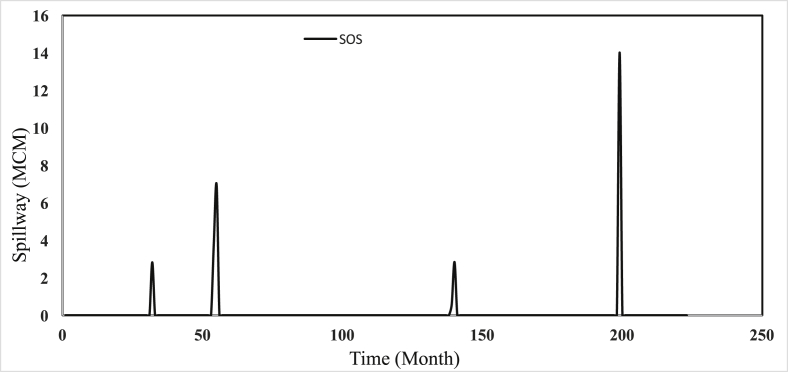
Water spillway patterns of SOS algorithm in Safarud Reservoir.

**Fig. 7:**
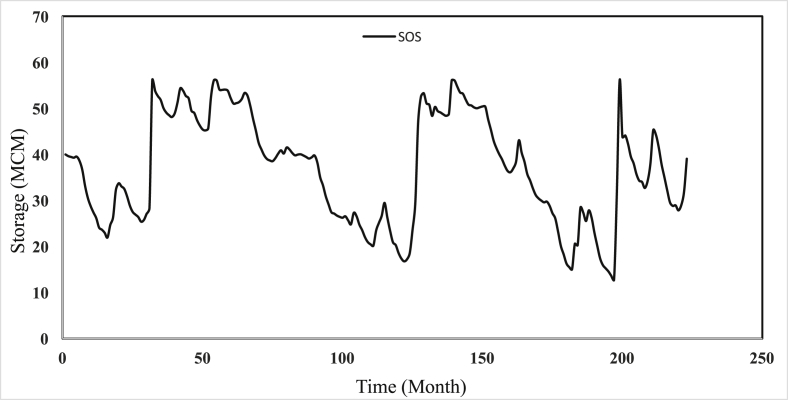
Water storage patterns of SOS algorithm in Safarud Reservoir.

Fig. 3 illustrates the geographical location of Safarud dam in Halilrood basin. Table 1 gives the characteristics of Safarud Reservoir, and Fig. 1 shows the image of Safarud dam. Moreover, Table 2 describes the values of SOS algorithm parameters for the reservoir operation problem. Fig. 4 displays the convergence rate of the SOS algorithm in reaching the optimum value for 100000 iterations. In addition, Fig. 5 depicts the release pattern for the operation of Safarud Reservoir for a period of 223 months. Fig. 6 shows the spillway pattern for the operation of Safarud Reservoir for a period of 223 months. Finally, Fig. 7 illustrates the storage pattern for the operation of Safarud Reservoir for this period.

**Table 1:** Characteristics of safarud reservoir.

Parameters	Unit	Value
Type of dam	–	Earth-dam
Dam height	m	72
Crest width	m	12
North latitude	Degree (°)	29° 15′
East longitude	Degree (°)	56° 58′
Minimum reservoir storage	MCM	17.3
Maximum reservoir storage	MCM	73.4
Minimum water demand	MCM	1.05
Maximum water demand	MCM	4.77
Normal level	MASL	2160

**Table 2:** Values of the SOS algorithm parameters for Safarud Reservoir operation.

Algorithm	Parameter	Iterations	Number of variables	Population size	Number of function evaluations	BF1	BF2
SOS	Value	100000	223	50	5,000,000	1 or 2	1 or 2

## 2 Experimental design, materials and methods

The SOS algorithm was applied for the optimal operation of Safarud Reservoir. The details of the SOS algorithm were provided by Cheng and Prayogo (2014) and Akbarifard and Radmanesh (2018) [1,2]. The SOS algorithm was coded in MATLAB software.

The optimization model for producing a time series dataset of minimizing the total deficit of Safarud Reservoir was structured in a monthly time step from 2000 to 2001 to 2018–2019. The optimum monthly release of the reservoir was considered as the decision variable, and the storage volume of the reservoir was regarded as the state variable. The total number of decision variables in this reservoir system was 223 (the number of time periods = 223 months), which is equal to the dimensions of the problem. The objective function was defined as Eq. (1) for Safarud Reservoir for the period of 2000–2019 (223 months).

(1) $$Minimize\;F\left(Re\right)=\sum_{t=1}^{T}{\left(\frac{De_{t}-Re_{t}}{De_{max}}\right)}^{2}+Penalty_{t}$$

where *F(Re)* is the objective function for Safarud Reservoir problem, Re_*t*_ is the release of the reservoir in the period of *t*, *De*_*t*_ denotes the downstream water demand of the reservoir in period *t*, *De*_*max*_ represents the maximum downstream demand of the reservoir during the entire operation period, *T* is the total number of operation periods of the reservoir, and *Penalty*_*t*_ is the penalty function related to the storage from the reservoir in period *t*. The penalty function related to the reservoir storage was defined as:

(2) $$Penalty_{t}=\{\begin{array}{c} \sum_{t=1}^{T}{\frac{\left(S_{t}-S_{\text{min}}\right)}{S_{\text{min}}}}^{2}\;if\;S_{t}<S_{\text{min}}\\ \sum_{t=1}^{T}{\frac{\left(S_{t}-S_{\text{max}}\right)}{S_{\text{max}}}}^{2}\;if\;S_{t}>S_{\text{max}}\\ 0\;\;if\;\;S_{t}\geq S_{\text{min}}\;\text{and}\;S_{t}\leq S_{\text{max}} \end{array}$$

Constraints of the reservoir are as follows:

(3) $$Sp_{t}=\{\begin{array}{c} S_{t}-S_{max}\;if\;S_{t}>S_{\text{max}}\\ 0\;\;\;\;if\;S_{t}\leq S_{\text{max}} \end{array}$$

(4) $$Loss_{t}=A_{t}\times \left(Ev_{t}-R_{t}\right)$$

(5) $$A_{t}=a+b\times S_{t}+c\times S_{t}^{2}$$

(6) $$S_{t+1}=S_{t}+Q_{t}-Re_{t}-Loss_{t}-Sp_{t}$$

(7) $$S_{\text{min}}\leq S_{t}\leq S_{\text{max}}$$

where *Sp*_*t*_ is the spill overflow from Safarud Reservoir in period *t*, *S*_*t*_ denotes the storage of the reservoir at the beginning of period *t*, *S*_max_ and *S*_min_ are the maximum and minimum of the storage of the reservoir, respectively, *Loss*_*t*_ indicates the loss from the reservoir in period *t*, *Ev*_*t*_ represents the depth of evaporation from the reservoir in period *t*, *R*_*t*_ is the depth of precipitation on the reservoir in period *t*, and *a, b,* and *c* are the coefficients of storage-surface relation for Safarud Reservoir. Moreover, *S*_*t+1*_ is the storage of Safarud Reservoir at the end of period *t,* and *Q*_*t*_ shows the inflow to the reservoir in period *t* [[3], [4], [5], [6], [7], [8], [9]].

### 2.1 Analysis of dataset

The analysis of the objective function revealed that the best solution achieved by the SOS algorithm for the reservoir was 10.89. Based on the analysis of these datasets, the MSA algorithm was a good algorithm for the optimal operation of Safarud Reservoir.

All analyses of this data article for the SOS algorithm are presented in Fig. 4, Fig. 5, Fig. 6, Fig. 7.
