# Supplementary material for: Data on optimal operation of Safarud Reservoir using symbiotic organisms search (SOS) algorithm
Source: Data Brief. 2020 Feb 26;29:105327. doi: 10.1016/j.dib.2020.105327 (PMC7078506; doi:10.1016/j.dib.2020.105327)
Supplement: Multimedia component 2 [file mmc2.pdf]

Virayeh

EDITORIAL CERTIFICATE

This document certifies that the manuscript with the following specifications was edited for proper English language, grammar, punctuation, spelling, and overall style by one or more of the highly qualified English language editors at Virayeh Editing & Translation Office.

MANUSCRIPT TITLE:

“Data on Optimal Operation of Safarud Reservoir Using Symbiotic Organisms Search (SOS) Algorithm”

AUTHORS:

Aliakbar Rezaei-Estakhroueiyeh, Navid Jalalkamali, Mehdi Momeniroghabadi

DATE ISSUED:

15-Feb-2020

CERTIFICATE VERIFICATION CODE:

ED10297.3547.16

www.virayeh.com
info@virayeh.com

This document certifies that the manuscript listed above was edited for proper English language and overall style by highly qualified English language editors at www.virayeh.com. Neither the research content, nor the authors' intentions were altered in any way during the editing process. Documents receiving this certification should be English-ready for publication; however, the author is able to accept or reject our suggestions and changes. To verify the final Virayeh-edited version, please visit our verification page: http://virayeh.com/view/generalpages/Certificate.php. If you have any questions or concerns about this edited document, feel free to contact us at info@virayeh.com.

Virayeh Paper Editing Service provides a range of editing and translation services for researchers around the world. Our top-quality editors possess the highest qualifications to edit research manuscripts written by non-native English speakers. For more information about our services, please visit www.virayeh.com.
